# Effects of Isosorbide Mononitrate and/or Cilostazol on Hematological Markers, Platelet Function, and Hemodynamics in Patients With Lacunar Ischaemic Stroke: Safety Data From the Lacunar Intervention-1 (LACI-1) Trial

**DOI:** 10.3389/fneur.2019.00723

**Published:** 2019-07-03

**Authors:** Jason P. Appleton, Gordon W. Blair, Katie Flaherty, Zhe Kang Law, Jane May, Lisa J. Woodhouse, Fergus Doubal, Nikola Sprigg, Philip M. Bath, Joanna M. Wardlaw

**Affiliations:** ^1^Stroke Trials Unit, Division of Clinical Neuroscience, University of Nottingham, Nottingham, United Kingdom; ^2^Stroke, Nottingham University Hospitals NHS Trust, Nottingham, United Kingdom; ^3^Brain Research Imaging Centre, Centre for Clinical Brain Sciences, University of Edinburgh, Edinburgh, United Kingdom; ^4^Edinburgh Dementia Research Centre in the UK Dementia Research Initiative, Edinburgh, United Kingdom; ^5^Edinburgh Imaging, University of Edinburgh, Edinburgh, United Kingdom; ^6^Department of Medicine, National University of Malaysia, Kuala Lumpur, Malaysia

**Keywords:** cilostazol, isosorbide mononitrate, blood pressure, safety, platelets, lacunar stroke, randomized clinical trial

## Abstract

**Background:** Cilostazol and isosorbide mononitrate (ISMN) are candidate treatments for cerebral small vessel disease and lacunar ischaemic stroke. As both drugs may influence hemoglobin and platelet count, and hemodynamics, we sought to assess their effects in the lacunar intervention-1 (LACI-1) trial.

**Methods:** Fifty-seven lacunar ischaemic stroke patients were randomized to immediate ISMN, cilostazol, or their combination for 9 weeks in addition to guideline stroke prevention. A fourth group received both drugs with a delayed start. Full blood count, platelet function, peripheral blood pressure (BP), heart rate and central hemodynamics (Augmentation index, Buckberg index) were measured at baseline, and weeks 3 and 8. Differences were assessed by multiple linear regression adjusted for baseline and key prognostic variables. Registration ISRCTN 12580546.

**Results:** At week 8, platelet count was higher with cilostazol vs. no cilostazol (mean difference, MD 35.73, 95% confidence intervals, 95% CI 2.81–68.66, *p* = 0.033), but no significant differences were noted for hemoglobin levels or platelet function. At week 8, BP did not differ between the treatment groups, whilst heart rate was higher in those taking cilostazol vs. no cilostazol (MD 6.42, 95% CI 1.17–11.68, *p* = 0.017). Buckberg index (subendocardial perfusion) was lower in those randomized to cilostazol vs. no cilostazol and in those randomized to both drugs vs. either drug. Whilst ISMN significantly increased unadjusted augmentation index (arterial stiffness, MD 21.19, 95% CI 9.08–33.31, *p* = 0.001), in isolation both drugs non-significantly reduced augmentation index adjusted for heart rate.

**Conclusions:** Cilostazol increased heart rate and platelet count, and reduced Buckberg index, whilst both drugs may individually reduce arterial stiffness adjusted for heart rate. Neither drug had clinically significant effects on hemoglobin or platelet function over 8 weeks. Further assessment of the safety and efficacy of these medications following lacunar ischaemic stroke is warranted.

## Introduction

Cerebral small vessel disease (SVD) is a common cause of stroke—both “lacunar” ischaemic and haemorrhagic stroke—cognitive impairment and dementia (1). SVD is a disorder of the small perforating arterioles of the brain involving endothelial dysfunction, inflammation (2), and blood-brain barrier breakdown (3). However, there are no specific treatments for either primary or secondary prevention of SVD and its associated clinical manifestations (4). Two medications with potential beneficial mechanisms of action have been highlighted for potential repurposing: cilostazol (a phosphodiesterase 3′ inhibitor used in stroke prevention in the Asia-Pacific Region) and isosorbide mononitrate (ISMN, a nitric oxide donor widely used in ischaemic heart disease). Little is known about the safety and efficacy of these drugs in SVD, particularly in combination, and yet their effects may be synergistic (4). As a mild antiplatelet, cilostazol can cause bleeding sufficient to result in anemia. Further, cilostazol can rarely cause thrombocythaemia (5). Both drugs can lower peripheral blood pressure (BP) and increase heart rate (5, 6), but data regarding their effects on central hemodynamics following ischaemic lacunar stroke are lacking.

The Lacunar Intervention Trial-1 (LACI-1) tested cilostazol and ISMN, alone and in combination, for tolerability in patients with prior lacunar ischaemic stroke (7). Overall, the medications were well-tolerated by participants (8). As both drugs may influence platelet count and hemoglobin level in addition to hemodynamics, we sought to assess these important safety outcomes.

## Methods

### Population

**Figure 1:**
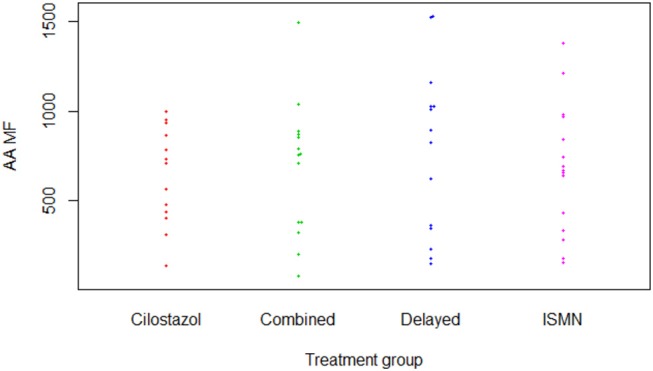
P-selectin surface expression in response to arachidonic acid (Aspirin test). AA, Arachidonic acid; ISMN, isosorbide mononitrate; MF, median fluorescence.

LACI-1 was a phase IIa, partial factorial, dose-escalation, prospective, randomized, open-label, blinded endpoint (PROBE) trial. Details regarding the trial protocol and statistical analysis plan are available (7), and the main results have been presented and published (8, 9). In summary, LACI-1 recruited 57 patients from stroke centers in Edinburgh and Nottingham, UK, with clinically confirmed lacunar ischaemic stroke, without dependency and able to consent themselves, and randomized them to one of four groups for 9 weeks: ISMN 25 mg twice daily; cilostazol 100 mg twice daily; both ISMN and cilostazol started immediately; or both medications with a 3-week delayed start, thus providing a drug-free control period (see Figure 1 in main paper) (8). Patients were contacted on alternate weeks to enquire about symptoms (headache, nausea, diarrhea, vomiting, and bleeding) using a structured questionnaire. All patients continued to take guideline secondary stroke prevention medications including statins and antihypertensive treatment where appropriate. Hematology and haemodynamic measures were assessed blind to treatment allocation.

All patients provided written informed consent. LACI-1 was approved by the Scotland A Research Ethics Committee (15/SS/0154) and registered (ISRCTN 12580546).

### Hematology and Platelet Function Testing

Full blood counts were taken at baseline, and at weeks 3 and 8: hemoglobin (g/L); and platelet count (×10^9^/L). Platelet function was assessed with measurement of surface expression of P-selectin (CD63P) using kits sensitive to aspirin (arachidonic acid) or clopidogrel (adenosine diphosphate) at the same timepoints (10). An unstimulated sample provided baseline expression data at each timepoint. Median fluorescence (MF) was recorded for platelet surface expression of P-selectin for each sample using flow cytometry. Platelet P-selectin expression was chosen since blood samples can be obtained and fixed at multiple clinical sites then transported for measurement at a central core laboratory.

### Haemodynamic Measures

Hemodynamics were measured at baseline and at weeks 3 and 8. Peripheral blood pressure (BP, mmHg) and heart rate (bpm) were measured three times sitting and three times standing at each visit using a validated monitor. Central hemodynamics were measured in duplicate using the SphygmoCor device at the same timepoints: mean arterial pressure (MAP); Augmentation index (%)—a measure of arterial stiffness; Buckberg index (%)—a measure of subendocardial perfusion; and pulse wave velocity (m/s)—a measure of arterial stiffness, assessed using non-invasive tonometry measurements taken at the radial and carotid arteries.

### Sample Size and Statistics

The sample size calculation for LACI-1 was based on the ability to detect a difference of 90 vs. 55% (i.e., an absolute difference of 35%) between those reaching target dose on one drug vs. both drugs. For 80% power, significance 0.05, a sample size of 55 was needed.

Data are number (%), mean (standard deviation, SD), and median [interquartile quartiles, IQR]. Baseline differences between treatment groups were assessed using chi-square and Kruskal-Wallis tests. Differences in hemodynamics and hematological measures were assessed by multiple linear regression, adjusted for baseline values, age, SVD imaging score and time to randomization. Haemodynamic measures were also adjusted for baseline systolic BP. As heart rate can lead to spurious results, Augmentation index (%) was measured unadjusted and normalized to heart rate 75 bpm as is standard practice. The resultant mean difference (MD) with 95% confidence intervals (CI) are presented with significance set at *p* <0.05. Analyses were performed using SAS.

## Results

In total, 57 patients were recruited at mean 203 [median 256] days after their index stroke with a mean age of 66 (11) years and 18 (32%) were female (Table 1). Most participants (97%) were on clopidogrel for stroke secondary prevention (guideline therapy in the UK), the remainder on aspirin. Apart from the cilostazol group being slightly older, baseline characteristics were well-balanced across the treatment groups. One participant in the delayed start group withdrew during follow-up.

**Table 1:** Baseline characteristics of LACI-1 participants.

	**All**	**Both delayed**	**ISMN**	**Cilostazol**	**Both immediate**
Patients	57	15	15	13	14
Age (years)^*^	66.1 (11.1)	63.4 (11.5)	62.2 (11.0)	75.8 (8.7)	64.4 (8.1)
Sex, female	18 (31.6%)	6 (40.0%)	4 (26.7%)	5 (38.5%)	3 (21.4%)
Onset to randomization (days)	202.6 (256.2)	153.3 (180.4)	137.5 (214.2)	279.2 (324.7)	254.0 (290.7)
Systolic BP (mmHg)	147.3 (20.5)	141.8 (19.6)	150.9 (19.0)	146.1 (21.3)	150.4 (22.9)
Diastolic BP (mmHg)	83.3 (12.6)	82.9 (14.3)	86.6 (11.5)	79.3 (12.0)	83.7 (12.6)
**Past medical history (%)**					
Treated hypertension	42 (73.7%)	11 (73.3%)	11 (73.3%)	12 (92.3%)	8 (57.1%)
Treated hyperlipidaemia	48 (84.2%)	11 (73.3%)	12 (80.0%)	13 (100.0%)	12 (85.7%)
Diabetes	11 (19.3%)	3 (20.0%)	3 (20.0%)	2 (15.4%)	3 (21.4%)
Atrial fibrillation	0	0	0	0	0
Myocardial infarction	1 (1.8%)	1 (6.7%)	0	0	0
Previous stroke	6 (10.5%)	1 (6.7%)	2 (13.3%)	1 (7.7%)	2 (14.3%)
Smoking	26 (45.6%)	10 (66.7%)	6 (40.0%)	6 (46.2%)	4 (28.6%)
**Patient status**					
mRS, baseline [/6]	1.0 [0.0, 1.0]	1.0 [0.0, 2.0]	1.0 [1.0, 1.0]	1.0 [1.0, 1.0]	1.0 [0.0, 1.0]
Current NIHSS [/42]	0.0 [0.0, 1.0]	0.0 [0.0, 1.0]	0.0 [0.0, 1.0]	0.0 [0.0, 1.0]	0.5 [0.0, 1.0]

BP, blood pressure; ISMN, isosorbide mononitrate; mRS, modified Rankin Scale; NIHSS, National Institute of Health Stroke Scale.

* *Significant difference between the treatment groups (p <0.05), comparisons done and Kruskal-Wallis tests*.

### Hematology and Platelet Function Testing

**Figure 2:**
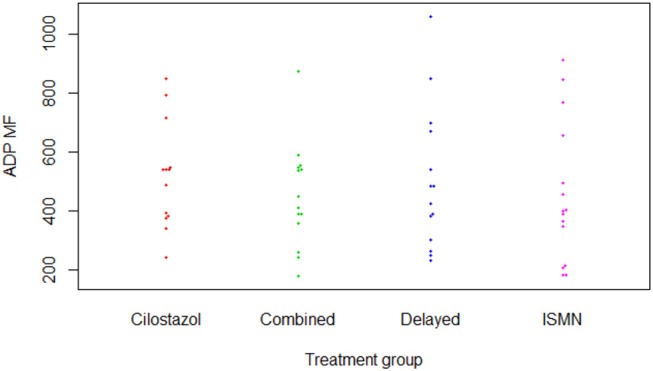
P-selectin surface expression in response to adenosine diphosphate (Clopidogrel test). ADP, adenosine diphosphate; ISMN, isosorbide mononitrate; MF, median fluorescence.

There were no differences in hemoglobin at week 8 between treatment groups (Table 2). Platelet count was slightly higher in those on cilostazol 286.8 ×10^9^/L vs. no cilostazol 249.1 ×10^9^/L (MD 35.73, 95% CI 2.81 to 68.66, *p* = 0.033). No differences were noted in platelet function between treatment groups in aspirin or clopidogrel tests (Figures 1, 2). There was no difference in bruising or bleeding with either medication in isolation or combination during the study.

**Table 2:** Hematology and platelet function testing at week 8 by treatment group.

**Week 8**	**Both delayed** ***N*** **= 14**		**ISMN *N* = 15**	**Cilostazol *N* = 13**	**Both immediate *N* = 14**	Cilostazol vs. none (2 + 3 vs. 1 + 4*)		ISMN vs. none (1 + 3 vs. 2 + 4*)		**Cil + ISMN vs. one or other (3 + 4 vs. 1 + 2)**	
**Full blood count, adjusted for baseline**	Week 3 (4*)	**Week 8 (4)**	**Week 8 (1)**	**Week 8 (2)**	**Week 8 (3)**	**MD (95% CI)**	***p*-value**	**MD (95% CI)**	***p*-value**	**MD (95% CI)**	***p*-value**
Hemoglobin (g/L), mean (SD)	133.0 (12.2)	136.2 (13.5)	146.9 (12.0)	130.8 (9.5)	137.1 (14.6)	−3.7 (−8.4, 1.0)	0.12	−1.0 (−6.9, 4.9)	0.73	−2.4 (−6.8, 2.1)	0.30
Platelet count (x10^9^/L), mean (SD)	260.3 (47.1)	258.4 (56.1)	238.6 (45.5)	289.5 (61.4)	284.3 (57.8)	35.7 (2.8, 68.7)	0.033	3.9 (−32.4, 40.2)	0.83	−7.4 (−36.8, 21.9)	0.62
**Platelet function, adjusted for baseline**											
Unstimulated median fluorescence, mean (SD)	87.0 (29.0)	80.4 (29.1)	86.1 (29.0)	75.9 (27.3)	83.7 (33.8)	−2.9 (−15.1, 9.3)	0.64	−8.2 (−20.7, 4.2)	0.20	7.5 (−4.0, 19.0)	0.20
AA median fluorescence, mean (SD)	741.0 (395.0)	775.4 (474.4)	675.2 (364.1)	636.4 (272.4)	679.6 (376.6)	−83.6 (−256.5, 89.4)	0.34	−14.1 (−192.5, 164.2)	0.88	109.7 (−54.0, 273.5)	0.19
ADP median fluorescence, mean (SD)	519.3 (234.5)	502.1 (242.8)	454.9 (238.8)	518.9 (179.5)	451.7 (176.5)	−1.3 (−77.6, 75.0)	0.97	−0.5 (−80.1, 79.1)	0.99	11.9 (−60.2, 84.1)	0.75

*AA, arachidonic acid (aspirin test); ADP, adenosine diphosphate (clopidogrel test); CI, confidence intervals; ISMN, isosorbide mononitrate; MD, mean difference; SD, standard deviation*.

### Hemodynamics

Full haemodynamic data were available for 56 participants. Baseline peripheral BP and heart rate did not differ between treatment groups (Table 1). At week 8, BP did not significantly differ between the treatment groups, whilst heart rate was significantly higher in those taking cilostazol 82.8 bpm vs. no cilostazol 74.5 bpm (MD 6.42, 95% CI 1.17 to 11.68, *p* = 0.017, Table 3). Central MAP did not differ between treatment groups at week 8. Buckberg index (subendocardial perfusion) was reduced in those randomized to cilostazol 142.2% vs. no cilostazol 160.7% (MD −10.81, 95% CI −21.15 to −0.47, *p* = 0.040) and in those randomized to both drugs 148.9% vs. either drug 159.9% (MD −11.37, 95% CI −21.01 to −1.72, *p* = 0.021). Unadjusted Augmentation index was significantly higher (i.e., increased arterial stiffness) in those randomized to ISMN 123.8% vs. no ISMN 119.8% (MD 21.19, 95% CI 9.08 to 33.31, *p* = 0.001). In contrast, when adjusted for heart rate there was a tendency toward lower Augmentation index (i.e., less arterial stiffness) in those randomized to cilostazol 18.7% vs. no cilostazol 20.6% (MD −4.79, 95% CI −9.72 to 0.15, *p* = 0.057) and ISMN 16.1% vs. no ISMN 23.5% (MD −4.74, 95% CI −9.78 to 0.30, *p* = 0.066). No differences in pulse wave velocity were seen.

**Table 3:** Peripheral and central hemodynamics by treatment group.

	**Both delayed** ***N*** **= 14**		**ISMN *N* = 15**	**Cilostazol *N* = 13**	**Both immediate *N* = 14**	Cilostazol vs. none (2 + 3 vs. 1 + 4*)		ISMN vs. none (1 + 3 vs. 2 + 4*)		**Cil + ISMN vs. one or other (3 + 4 vs. 1 + 2)**	
**Peripheral hemodynamics, adjusted for baseline**	Week 3 (4*)	**Week 8 (4)**	**Week 8 (1)**	**Week 8 (2)**	**Week 8 (3)**	**MD (95% CI)**	***p*-value**	**MD (95% CI)**	***p*-value**	**MD (95% CI)**	***p*-value**
Systolic (mmHg), mean (SD)	134.8 (12.4)	131.4 (19.0)	144.1 (16.5)	146.7 (19.4)	139.9 (20.7)	−1.11 (−8.38, 6.16)	0.765	−1.06 (−8.52, 6.40)	0.780	−4.91 (−11.71, 1.88)	0.156
Diastolic (mmHg), mean (SD)	80.4 (9.7)	78.0 (10.8)	84.9 (7.6)	81.3 (11.2)	79.8 (10.5)	−1.63 (−5.84, 2.58)	0.448	−0.65 (−4.96, 3.66)	0.768	−2.56 (−6.50, 1.38)	0.202
Heart rate, mean (SD)	74.3 (9.3)	79.5 (11.2)	74.7 (12.5)	80.6 (15.0)	84.8 (15.1)	6.42 (1.17, 11.68)	0.017	0.06 (−5.50, 5.62)	0.984	3.13 (−1.93, 8.20)	0.225
**Central hemodynamics, adjusted for baseline**											
Pulse wave velocity (m/s), mean (SD)	8.4 (1.3)	8.7 (1.4)	8.7 (1.6)	8.6 (1.3)	8.2 (1.3)	−0.34 (−0.99, 0.30)	0.296	−0.14 (−0.84, 0.55)	0.686	0.00 (−0.64, 0.64)	0.996
Augmentation index 75 (%), mean (SD)	24.0 (10.6)	16.2 (12.4)	17.5 (12.9)	23.0 (10.6)	14.7 (10.5)	−4.79 (−9.72, 0.15)	0.057	−4.74 (−9.78, 0.30)	0.066	0.09 (−4.76, 4.94)	0.970
Augmentation index (%), mean (SD)	118.6 (28.3)	123.3 (23.2)	131.1 (16.8)	121.1 (18.9)	133.1 (25.0)	3.46 (−8.65, 15.57)	0.575	21.19 (9.08, 33.31)	0.001	0.97 (−10.77, 12.71)	0.871
Central blood pressure (mmHg), mean (SD)	99.0 (14.6)	95.4 (12.7)	101.2 (12.4)	103.1 (11.8)	98.8 (11.7)	−2.03 (−8.23, 4.17)	0.521	−4.74 (−10.67, 1.19)	0.117	−3.36 (−9.12, 2.40)	0.253
Buckberg index (%), mean (SD)	154.4 (21.5)	164.8 (22.2)	166.6 (25.2)	152.2 (31.4)	132.9 (22.7)	−10.81 (−21.15, −0.47)	0.040	6.75 (−3.59, 17.09)	0.201	−11.37 (−21.01, −1.72)	0.021

*CI, confidence intervals; ISMN, isosorbide mononitrate; MD, mean difference; SD, standard deviation. Augmentation index 75: normalized to heart rate 75 bpm*.

## Discussion

In this secondary analysis of the LACI-1 trial—the first trial to assess cilostazol and/or ISMN in lacunar ischaemic stroke patients, and the first trial to assess both together in any ischaemic stroke—we have demonstrated that cilostazol and ISMN in isolation and in combination do not influence hemoglobin levels or platelet function in the short-term when given in addition to usual secondary stroke prevention therapy. Platelet count was significantly higher in those randomized to cilostazol. Cilostazol alone and both drugs in combination, reduced Buckberg index (subendocardial perfusion), whilst cilostazol increased heart rate. Both drugs in isolation may reduce arterial stiffness when adjusted for heart rate.

Due to its mild antiplatelet effects, cilostazol can uncommonly (≥1/100 to <1/10) cause bleeding sufficient to lead to anemia according to the summary of product characteristics (SmPC) (5). Although we have a small population, it is reassuring that hemoglobin was stable between treatment groups during the trial given that all participants were already on guideline antiplatelet therapy. The increase in platelet count seen with cilostazol at 8 weeks needs further investigation, especially since thrombocythaemia, is known to occur rarely (≥1/10,000 to <1/1,000) (5). The lack of any difference in platelet function between the treatment groups is of further reassurance.

ISMN is known to lower BP and increase heart rate and so can commonly lead to postural dizziness (≥1/100 to <1/10) and rarely syncope (6). Cilostazol can increase heart rate and, if co-administered with BP-lowering medication, an additive hypotensive effect with reflex tachycardia is reported (5). Neither symptom were apparent across the trial population (8). Importantly, despite cilostazol increasing heart rate in our population, no significant hypotensive effect was seen in combination with participants' pre-prescribed antihypertensives (74% were on antihypertensives at randomization) or ISMN. Central haemodynamic data regarding cilostazol and/or ISMN in the context of lacunar ischaemic stroke are lacking. Buckberg index is a marker of subendocardial perfusion, which is reduced with increased heart rate (11). Cilostazol and both drugs in combination reduced Buckberg index, which may be due to their underlying effects on heart rate, or may reflect that the patients allocated to cilostazol were older than those in other allocated groups. ISMN was associated with increased Augmentation index (a marker of arterial stiffness) unadjusted for heart rate, but when Augmentation index was adjusted for heart rate, cilostazol and ISMN in isolation both non-significantly reduced arterial stiffness. Given that small vessel stiffness is implicated in the pathophysiology of cerebral SVD (12), medications that reduce arterial stiffness may be of benefit.

The strengths of this LACI-1 substudy include using data from the first randomized controlled trial assessing both cilostazol and ISMN in lacunar ischaemic stroke patients. There are, however, limitations. First, the sample size was small and the study was not powered to look for differences in hematological or haemodynamic secondary outcomes, therefore some findings may represent chance. Second, the treatment duration was relatively short and so we cannot comment on the longer-term effects of these medications on hematological markers or hemodynamics. Third, those who were randomized to cilostazol were older which may explain some of the reported differences in these outcomes. Last, there was no formal control group throughout the trial, rather the delayed start group represented a drug-free control period. This gave the opportunity to establish whether starting one drug first over the other impacted upon side-effects, whilst still providing a control group for the first 3 weeks of the study.

In summary, cilostazol and ISMN were safe in isolation and combination in the short- to medium-term in this small population following lacunar ischaemic stroke. Further data from larger trials are required to establish the longer term effects of these medications on safety and efficacy following lacunar ischaemic stroke. As such, the LACI-2 study is testing their effects on stroke recurrence, cognitive function, imaging features of SVD and safety when given for 12 months in patients with lacunar ischaemic stroke (ISRCTN14911850).

## Data Availability

The datasets generated for this study are available on request to the corresponding author.

## Ethics Statement

LACI-1 was approved by the Scotland A Research Ethics Committee (15/SS/0154) and registered (ISRCTN 12580546).

## Author Contributions

JA: recruitment, data collection, analysis, and manuscript preparation. GB and ZL: recruitment, data collection, analysis, and manuscript editing. KF and LW: statistical analysis, manuscript preparation, and editing. JM: platelet function testing. FD and NS: study design, supervision, study set up, data collection, SAE adjudication, and manuscript editing. PB: trial conception, design, management, supervision, data collection and analysis, and manuscript editing. JW: trial conception, design, funding, supervision, data collection, analysis, manuscript editing, and overall guarantor.

### Conflict of Interest Statement

The authors declare that the research was conducted in the absence of any commercial or financial relationships that could be construed as a potential conflict of interest.

## Abbreviations

Bpm: beats per minute
BP: blood pressure
CI: confidence interval
FBC: full blood count
ISMN: isosorbide mononitrate
MD: mean difference
SVD: small vessel disease.
